# Recurrent Spontaneous Pneumothorax in a Nine-Year-Old Patient

**DOI:** 10.7759/cureus.99775

**Published:** 2025-12-21

**Authors:** Mahmoud Yehya, Adel Taha, Alyaa Alramah, Rigashy Raghavan, Maaida Sheikh

**Affiliations:** 1 Thoracic Surgery, Saqr Hospital - Emirates Health Services, Ras Al Khaimah, ARE; 2 Intensive Care Unit, Saqr Hospital - Emirates Health Services, Ras Al Khaimah, ARE; 3 General Physician, Ras Al Khaimah Medical and Health Sciences University, Ras Al Khaimah, ARE; 4 General Physician, University Hospital, Sharjah, ARE

**Keywords:** chest tube drainage, pediatric pneumothorax, pleurodesis, primary spontaneous pneumothorax, pulmonary bullae, recurrent pneumothorax, thoracoscopic bullectomy, video-assisted thoracoscopic surgery (vats)

## Abstract

Spontaneous pneumothorax is defined as lung collapse due to air accumulation in the pleural space without trauma or iatrogenic causes and is rare in the pediatric population, with an incidence of approximately 1-4 per 100,000 children. Management strategies are largely extrapolated from adult guidelines, and pediatric evidence is primarily limited to case reports. We report a case of a nine-year-old boy who presented with sudden-onset pleuritic chest pain and was diagnosed with a large right-sided spontaneous pneumothorax. Initial management involved chest tube insertion, and subsequent computed tomography revealed right-sided congenital bullae located in the apical segment of the right lower lobe. Ten days later, the patient developed an early ipsilateral recurrence, prompting video-assisted thoracoscopic surgery with bullectomy and pleurodesis. This case highlights the challenges of managing pediatric spontaneous pneumothorax in the absence of standardized pediatric guidelines and suggests that early imaging and individualized consideration of surgical intervention may help reduce recurrence risk. The development of pediatric-specific management guidelines is warranted.

## Introduction

Pneumothorax occurs when air enters the pleural space, increasing intrathoracic pressure and causing partial or complete lung collapse.

Pneumothorax is categorized as spontaneous, iatrogenic, or traumatic. Spontaneous pneumothorax is further classified as primary, occurring secondary to blebs or bullae in otherwise healthy lungs, and secondary, occurring in association with underlying acute or chronic lung diseases [1,2].

Patients typically present with a sudden onset of sharp chest pain that worsens with deep inspiration or coughing, accompanied by shortness of breath. This condition is more prevalent in adults, with an annual incidence ranging from 7.4 per 100,000 individuals in the United States to 37 per 100,000 in the United Kingdom [1]. In contrast, the reported incidence in the pediatric population is approximately 4 per 100,000 males and 1.1 per 100,000 females per year, indicating a significantly lower occurrence in children [2-4]. Due to its rarity in pediatric cases, current management guidelines are primarily based on adult data.

In clinical practice, accurate diagnosis is the first step toward management. As per European Respiratory Society (ERS) recommendations, a posterior-anterior chest radiograph is the standard initial investigation for suspected primary spontaneous pneumothorax (PSP). When further assessment is required, computed tomography (CT) provides valuable cross-sectional imaging, particularly in complicated cases, suspected tube malposition, underlying lung pathology, or when surgical intervention is being considered [5].

Management approaches differ slightly among major adult guidelines. For adults, the British Thoracic Society (BTS) recommends needle aspiration as the first-line treatment for sizable or symptomatic primary pneumothorax, whereas the American College of Chest Physicians (ACCP) advises chest tube insertion when the pneumothorax occupies more than 20% of the hemithorax, regardless of symptom severity [5,6]. In contrast, pediatric management lacks standardized evidence-based guidelines due to the rarity of the condition. Current practice in children is largely extrapolated from adult protocols and accumulated experience from reported cases rather than formal consensus statements. Furthermore, there is no clear recommendation regarding early elective surgical intervention, such as pleurodesis or bullectomy, before the first recurrence; most guidelines emphasize definitive treatment only after recurrence [5].

## Case presentation

A nine-year-old boy presented with a sudden onset of sharp, right-sided pleuritic chest pain, preceded by a two-week history of persistent cough following an upper respiratory infection. He denied any recent history of trauma or vigorous physical activity.

On examination, the patient appeared anxious and tachypneic, in mild respiratory distress, but was otherwise hemodynamically stable. Inspection revealed asymmetrical chest expansion with reduced movement of the right hemithorax. Percussion demonstrated hyperresonance on the right side, and auscultation demonstrated absent breath sounds over the same area. Examination of other systems was unremarkable. 

Initial laboratory evaluation revealed normal hematological parameters, including white blood cell count, hemoglobin, hematocrit, and platelet count. However, inflammatory markers were mildly elevated, with C-reactive protein and erythrocyte sedimentation rate above the reference range, suggesting an underlying inflammatory process. Laboratory findings are summarized in Table 1. 

**Table 1:** Laboratory findings with reference ranges CRP and ESR are elevated, indicating an inflammatory process, while other hematological parameters remain within normal limits. CRP, C-reactive protein; ESR, erythrocyte sedimentation rate.

Test name	Patient result	Normal low	Normal high
White blood cell	12.14×10³/µL	5.00	13.00
Hemoglobin	13.1 g/dL	11.5	15.5
Hematocrit	37.8%	35.0	45.0
Platelet count	361×10³/µL	170.00	450.00
C-reactive protein	11.9 mg/L	0.0	3.0
Erythrocyte sedimentation rate	20 mm/hr	0.00	15.00

**Figure 1:**
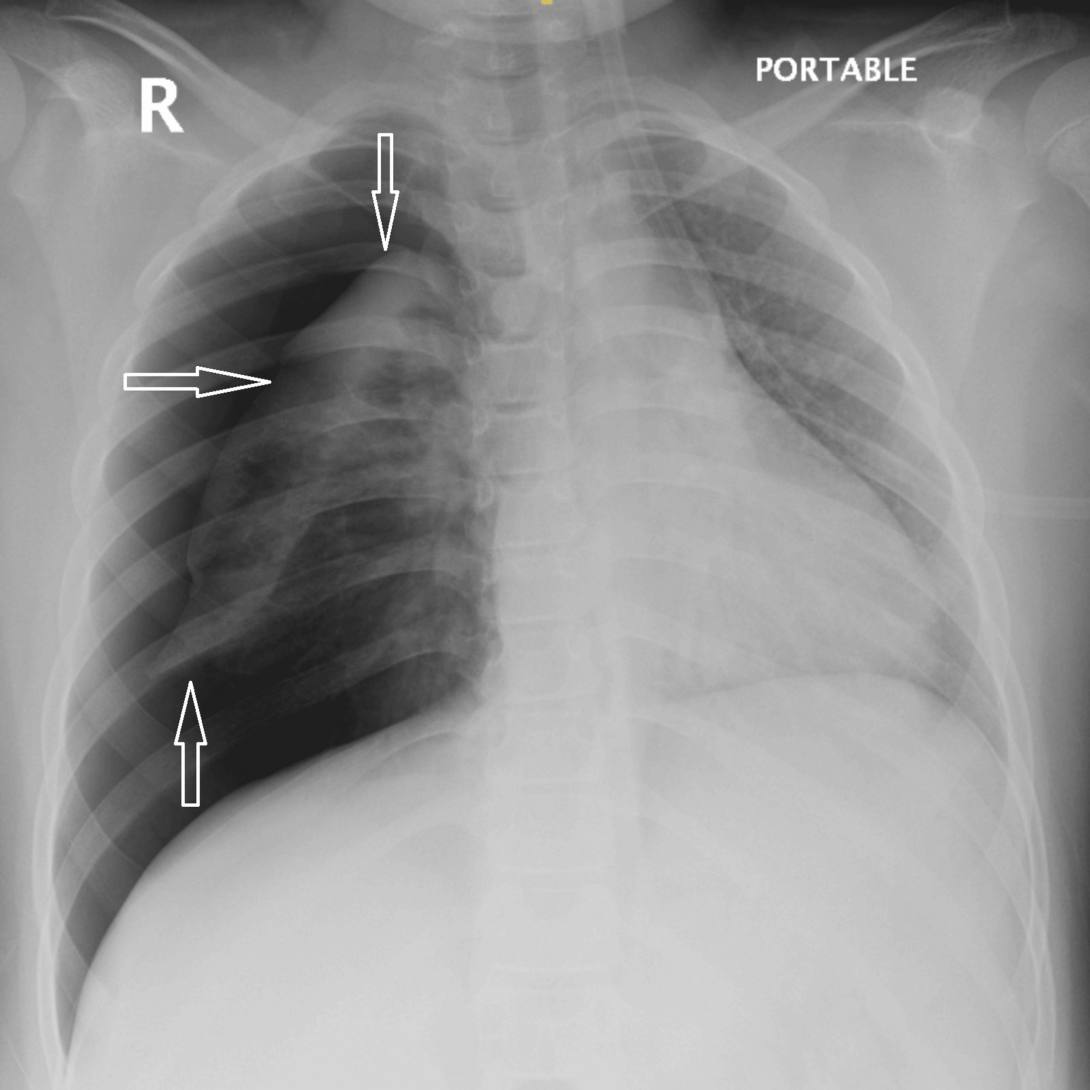
Chest radiograph of right-sided pneumothorax Chest X-ray demonstrating a marked right-sided pneumothorax with subtotal lung collapse. Arrows indicate the collapsed lung margin surrounded by the thin visceral pleura, and the pleural air space is clearly visible.

A plain chest radiograph demonstrated a marked right-sided pneumothorax (Figure 1).

**Figure 2:**
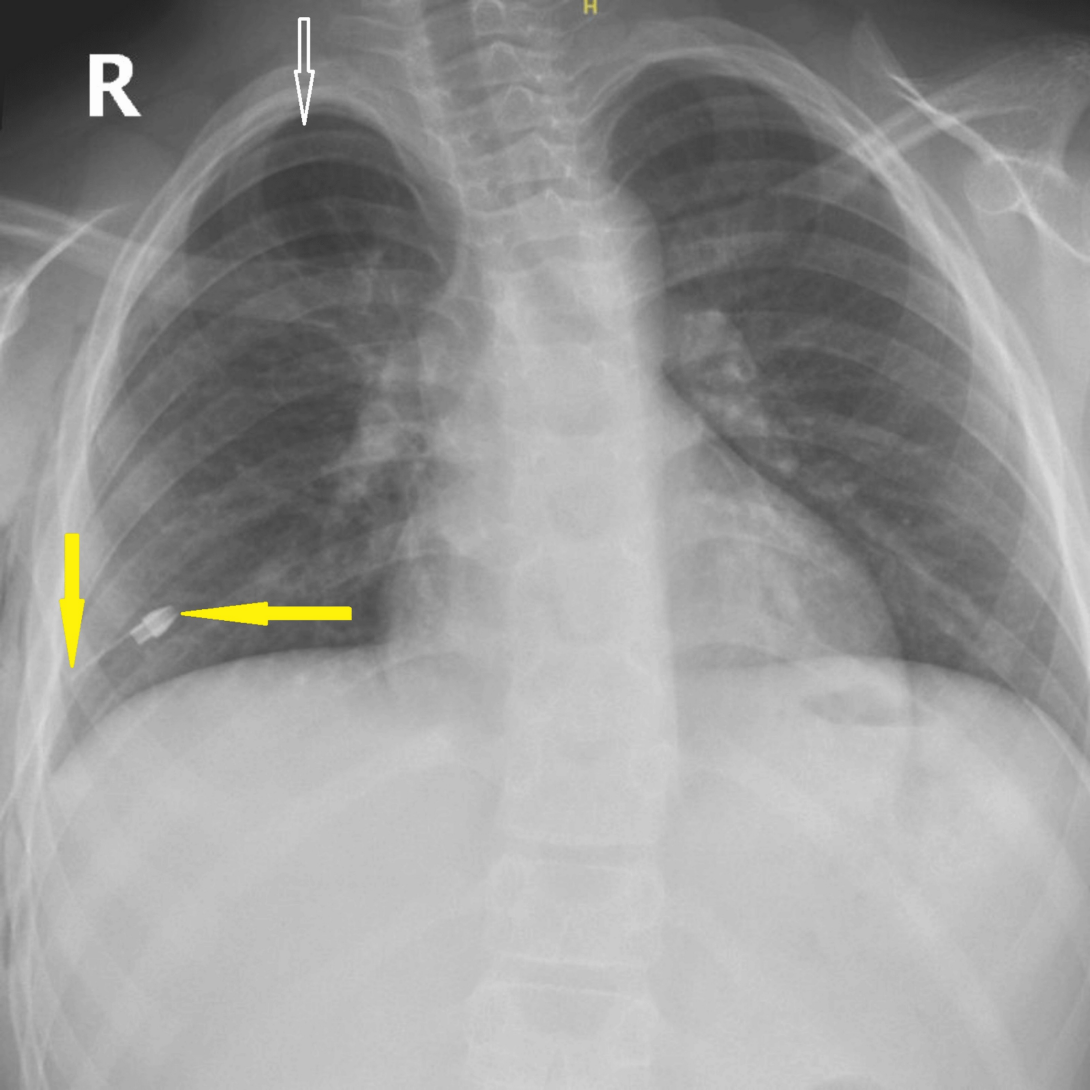
Post-drainage chest radiograph Chest X-ray showing re-expansion of the right lung following chest tube insertion. Solid yellow arrows indicate the chest tube position, and the hollow white arrow indicates a small residual apical pneumothorax pocket, which resolved later during follow-up. The tube side holes were confirmed to be within the thoracic cavity on subsequent CT imaging.

The patient was diagnosed with a sizable spontaneous pneumothorax. In the absence of standardized pediatric guidelines, chest tube insertion was performed in line with adult recommendations, which advocate immediate drainage for large or symptomatic pneumothorax to ensure rapid lung re-expansion and reduce the risk of complications. An 18-French chest tube was inserted in the right fifth intercostal space at the anterior axillary line. A small residual pocket of apical pneumothorax was noted on the post-drainage chest radiograph (Figure 2). However, this did not affect clinical management and was resolved completely during follow-up. 

**Figure 3:**
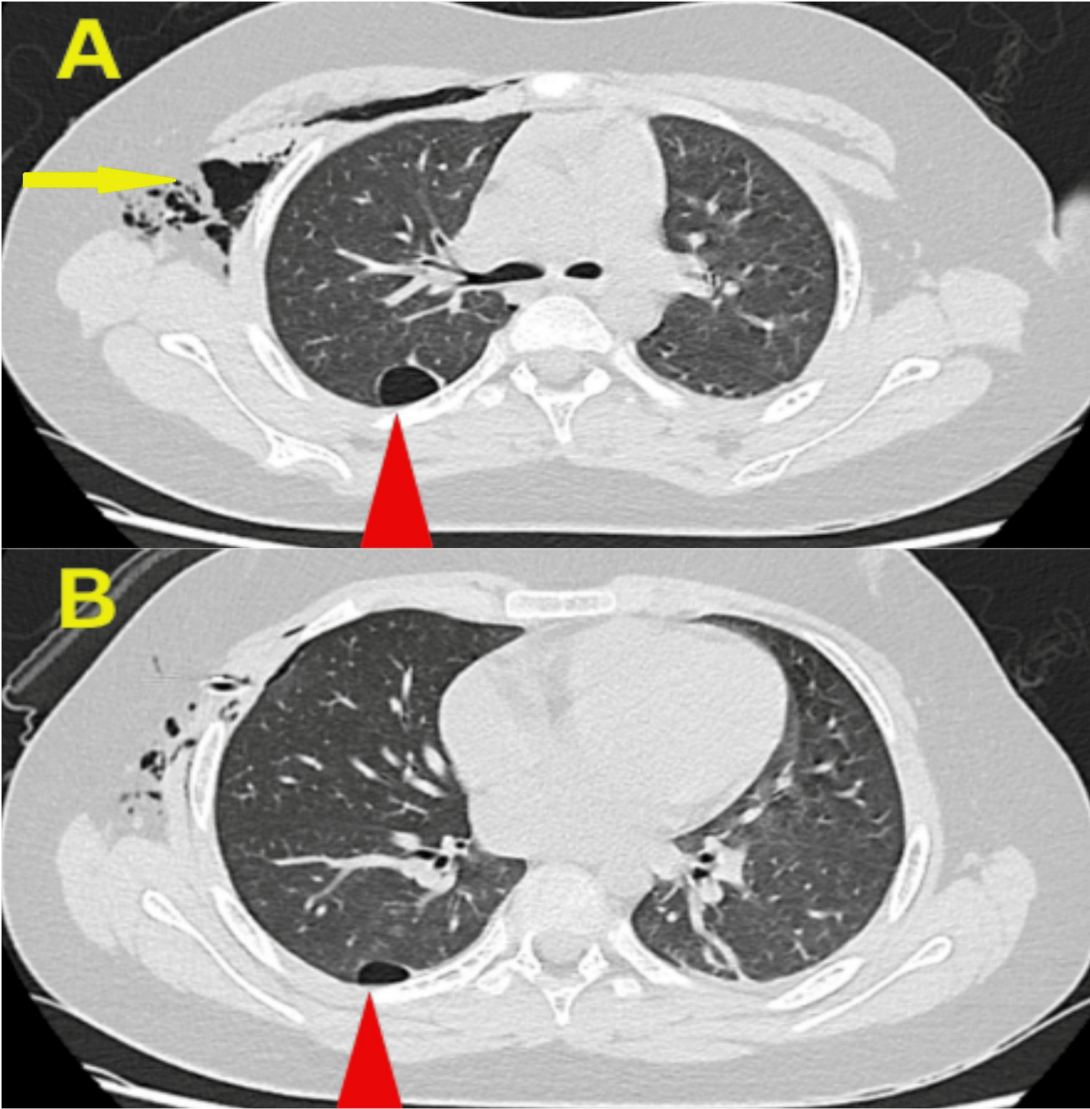
Post-drainage chest CT (A) Peripheral cystic lesion with a thin wall in the apical segment of the right lower lobe, consistent with a bulla (red arrow), along with post-drainage subcutaneous emphysema (yellow arrow). (B) A smaller bulla is visible in the same segment (red arrow). CT, computed tomography.

CT of the chest revealed two cystic lesions in the apical segment of the right lower lobe of the lung, consistent with pulmonary bullae (Figure 3).

The chest drain was removed on day 3, and the patient was discharged in stable condition.

**Figure 4:**
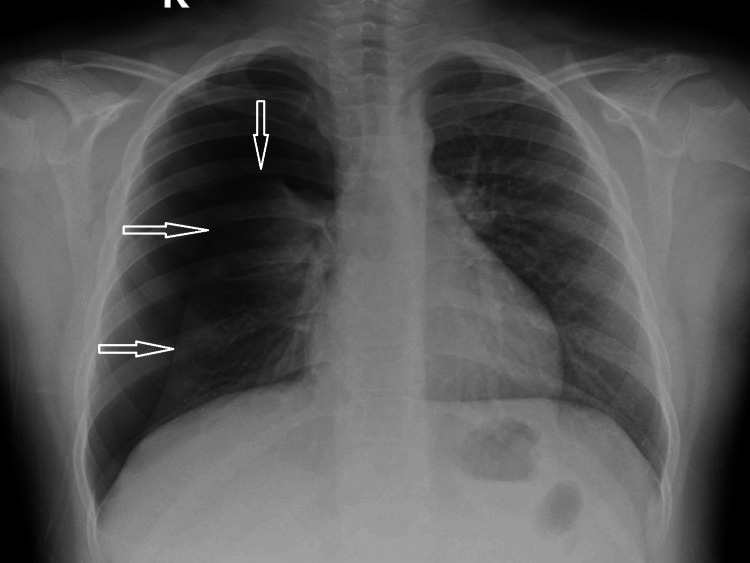
Chest radiograph showing recurrent ipsilateral pneumothorax Chest X-ray demonstrating recurrent right-sided pneumothorax. Hollow white arrows indicate the margins of the collapsed lung.

Ten days later, he returned with a dry cough and mild desaturation (oxygen saturation 92%-93% on room air). Imaging confirmed a recurrent ipsilateral pneumothorax (Figure 4).

As the patient remained hemodynamically stable with adequate oxygenation, chest drainage was deferred, and definite surgical management was planned.

**Figure 5:**
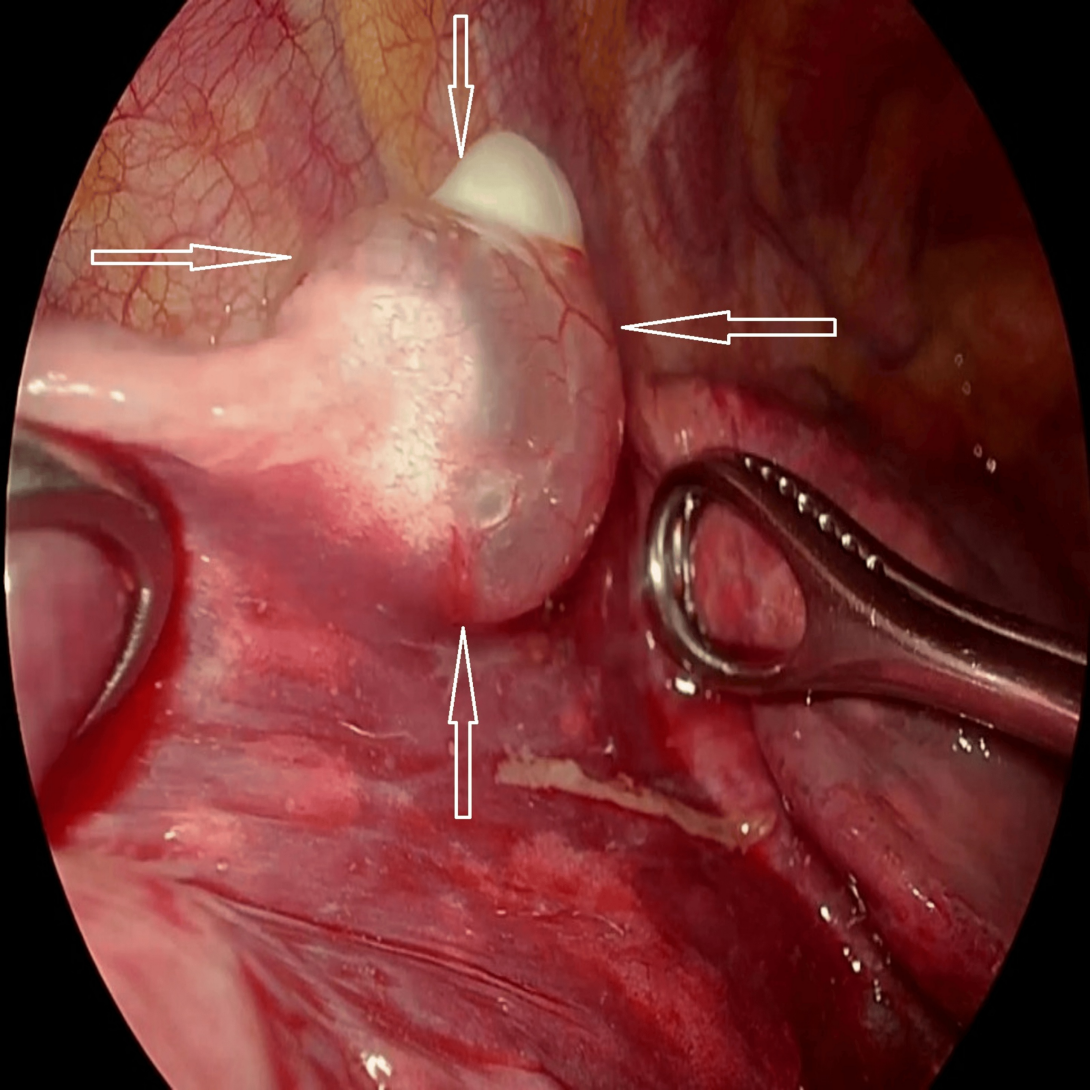
Intraoperative view during VATS showing bullae in the right lower lobe VATS image demonstrating one of two peripheral bullae (indicated by hollow white arrows) located in the apical segment of the right lower lobe. Bullectomy and pleurodesis were performed as definitive management. VATS, video-assisted thoracoscopic surgery.

Video-assisted thoracoscopic surgery (VATS) identified two peripheral bullae located in the apical segment of the right lower lobe (Figure 5).

**Figure 6:**
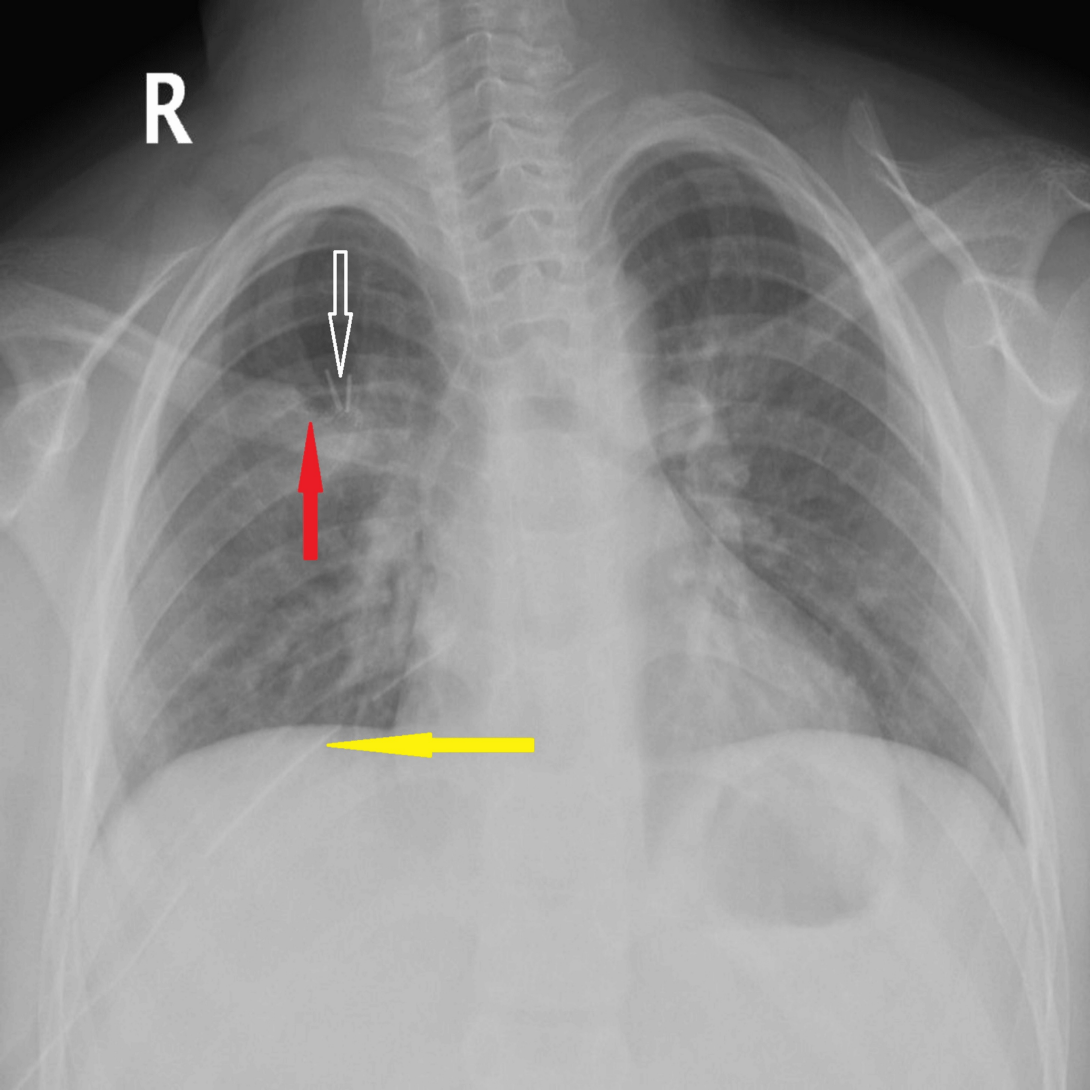
Postoperative chest radiograph demonstrating complete right lung re-expansion Postoperative chest X-ray showing complete re-expansion of the right lung following surgical intervention. Key features are indicated with arrows: Yellow arrow (left): Chest tube in situ Red arrow (up): Stapler line from bullectomy Wide hollow arrow: Endoscopic clips used during pleurodesis

 Bullectomy and surgical pleurodesis were performed. The procedure was performed under general anesthesia with single-lung ventilation using a 28-French double-lumen endotracheal tube. Postoperatively, pain control included intercostal nerve infiltration with a total of 20 mg combination of lidocaine and bupivacaine (Marcaine) administered at the end of the procedure during surgical closure, parenteral paracetamol and nalbuphine during the immediate postoperative period for 48 hours, followed by oral analgesics. This multimodal analgesic regimen provided effective pain control and facilitated early mobilization. A postoperative chest radiograph demonstrated complete lung re-expansion (Figure 6).

The postoperative course was uneventful; the chest drain was removed on postoperative day 3, and the patient was discharged home in good condition.

## Discussion

Clinical presentation

Spontaneous pneumothorax is a rare pathology in the age group between neonatal and adolescence. While some patients may be asymptomatic, the typical presentation includes dyspnea, tachypnoea, tachycardia, sharp chest pain, and hypoxia. Once a diagnosis of spontaneous pneumothorax is confirmed, prompt intervention is required, ranging from supplemental oxygen to chest tube insertion. The choice of intervention depends on whether the pneumothorax is primary versus secondary, its size, vital signs, and overall clinical presentation [2].

In this nine-year-old patient, the condition occurred in a relatively rare age group, with no prior history of respiratory disease. He presented with sharp pleuritic chest pain, tachypnoea, and tachycardia following a two-week history of persistent cough. This presentation is consistent with PSP, likely related to intrathoracic pressure changes during extensive coughing fits. Similar presentations have been previously reported in pediatric patients, where intense or prolonged coughing was implicated in spontaneous pneumothorax. For example, Bhattacharya et al. described an infant who developed spontaneous pneumothorax after a two-week pertussis-related cough, likely due to bleb rupture during sustained coughing strain [7]. These cases highlight that even in structurally normal lungs, cough-induced pressure spikes can trigger pneumothorax in the pediatric population. Chest X-ray was the first-line investigation, revealing a significant right-sided pneumothorax, and a small-bore thoracostomy drain was inserted.

Management and complications

Recurrence is the most common complication in children managed non-surgically after a first episode, occurring in 32%-50% of cases [3,8]. A recent meta-analysis of 2,475 adult patients found that patients with abnormal CT findings, including blebs or bullae, were 2.5 times more likely to experience recurrence than those without. Despite this, current clinical pathways do not recommend routine thoracic CT in all cases of spontaneous pneumothorax [9]. There is consensus that definitive treatment should be offered to patients with recurrence [5]. Historically, Sattler first identified bullae on the visceral pleura and linked them to pneumothorax [10]. While surgical bullectomy has been considered standard treatment, subsequent studies, including Janssen et al., found no increased incidence of bullae in recurrent PSP, suggesting they may not be a major risk factor [11]. Consistent with current guidelines, the BTS recommends surgical pleurodesis and/or bullectomy for adults with recurrent spontaneous pneumothorax [6].

Surgical considerations

In our patient, due to the absence of standardized pediatric guidelines for spontaneous pneumothorax, initial management decisions, including chest tube insertion, were guided by adult protocols, which recommend immediate drainage for large or symptomatic pneumothoraces to prevent complications. A post-drainage CT was performed to evaluate for underlying structural abnormalities, such as bullae or blebs, which are known risk factors for recurrence and influence decisions regarding definitive management [5,9]. The scan revealed two bullae in the apical segment of the right lower lobe. Given the lack of randomized evidence supporting prophylactic surgical management after a first episode, we initially opted for non-surgical management and discharged the patient. However, he returned 10 days later with an early ipsilateral recurrence, after which surgical intervention was performed using the standard three-port VATS approach with excellent outcomes. While uniportal VATS is increasingly used to minimize surgical trauma and improve cosmesis, current evidence does not establish its superiority over the conventional three-port technique in recurrent spontaneous pneumothorax cases [12]. This case underscores several important considerations for clinical practice.

Implications and recommendations

Although surgical intervention could have been considered upon identification of bullae to prevent recurrence, current adult guidelines recommend surgery primarily for recurrent cases. This highlights the absence of clear pediatric-specific guidelines. There is a need for pediatric-specific management protocols for spontaneous pneumothorax, which could include performing a chest CT scan during the initial episode to identify underlying pathology such as bullae, and considering early surgical intervention when bullae are present to reduce the risk of recurrence.

These recommendations should be further investigated and validated through comprehensive clinical studies.

## Conclusions

Pediatric spontaneous pneumothorax is uncommon but potentially life-threatening, requiring prompt recognition and individualized management. Given the high recurrence rate in children, clinicians should consider performing a chest CT during the initial episode to identify underlying abnormalities such as bullae. When bullae are present, early surgical intervention may help reduce recurrence risk and improve outcomes. Emerging technologies, including predictive imaging, which uses advanced imaging techniques to anticipate disease progression or risk of recurrence, and risk stratification tools may further enhance individualized care in the future. These strategies underscore the urgent need for pediatric-specific guidelines and warrant validation through comprehensive clinical studies.
